# A Review on Strength and Durability Properties of Wooden Ash Based Concrete

**DOI:** 10.3390/ma15207282

**Published:** 2022-10-18

**Authors:** Buthainah Nawaf AL-Kharabsheh, Mohamed Moafak Arbili, Ali Majdi, Jawad Ahmad, Ahmed Farouk Deifalla, A. Hakamy

**Affiliations:** 1 Department of Civil Engineering, Faculty of Engineering, Al-Albayt University, Al-Mafraq 25113, Jordan; 2 Department of Information Technology, Choman Technical Institute, Erbil Polytechnic University, Erbil 44001, Iraq; 3 Department of Building and Construction Techniques Engineering, Al-Mustaqbal University College, Hillah 51001, Iraq; 4 Department of Civil Engineering, Military College of Engineering, Sub Campus of National University of Sciences and Technology, Islamabad 44000, Pakistan; 5 Structural Engineering Department, Faculty of Engineering and Technology, Future University in Egypt, New Cairo 11845, Egypt; 6 Department of Physics, Faculty of Applied Science, Umm Al-Qura University, Makkah 21955, Saudi Arabia

**Keywords:** concrete, wooden ash, setting time, compressive strength, and acid attacks

## Abstract

The partial replacement of cement in concrete with other building materials has come to light because of research on industrial waste and sustainable building practices. Concrete is made more affordable by using such components, and it also helps to ease disposal worries. Ash made by burning wood and other wood products is one example of such a substance. Many researchers focused on the utilization of wooden ash (WA) as a construction material. However, information is scattered, and no one can easily judge the impact of WA on concrete properties which restrict its use. Therefore, a details review is required which collect the past and current progress on WA as a construction material. relevant information. This review aims to collect all the relevant information including the general back of WA, physical and chemical aspects of WA, the impact of WA on concrete fresh properties, strength properties, and durability aspects in addition to microstructure analysis. The results indicate the WA decreased the slump and increased the setting time. Strength and durability properties improved with the substitution of WA due to pozzolanic reaction and micro-filling effects. However, the optimum dose is important. Different research recommends different optimum doses depending on source mix design etc. However, the majority of researcher suggests a 10% optimum substitution of WA. The review also concludes that, although WA has the potential to be used as a concrete ingredient but less researchers focused on WA as compared to other waste materials such as fly ash and silica fume etc.

## 1. Introduction

Since carbon dioxide emissions are rising at an unprecedented pace, the anticipated global warming by environmental experts over the previous several decades is already a reality [1,2,3]. Approximately 7% of all greenhouse gas emissions come from the cement industry, which heavily contributes to this problem. Carbon dioxide is released into the environment at a rate of one ton of cement produced [4,5]. The carbon dioxide emissions are predicted to increase by 50% from present levels by the year 2020. Each year, the energy-intensive process needed to make Portland cement results in the emission of around 13,500 million tons of greenhouse gases [6].

It is common knowledge that cement is an expensive substance and that in order to produce it, limestone, a natural resource, must be used [7,8,9]. When cement is produced, a pollutant called CO_2_ is released in enormous amounts [10,11,12]. The availability of energy sources such as coal and oil is declining since they are utilized to make cement. Researchers are looking for readily accessible and affordable pozzolanic materials in the sector [13,14,15,16]. A study also conclude that it is important to use alternative materials instead of cement [17].

Several scholars are now expanding their study in the fields of global environmental conservation and the use of renewable resources [18,19,20]. Recent studies have concentrated on controlling building costs and using industrial waste as a partial substitute for cement. Several studies have looked at the qualities of freshly laid and cured concrete by partly substituting industrial byproducts for cement [21,22,23]. Among these byproducts, wood ash offers hopeful substitutes for cement that are only partially effective [24]. According to research, mortars that have been altered with WA are appropriate for use on cement composites because they have sufficient physicochemical qualities. Additionally, the addition of the ashes has a favorable impact from a sustainability perspective [25].

The average quantity of ash created by wood burning is 6 to 10%of the total weight of the wood burnt, and the makeup of this ash may vary greatly depending on the environment and the kind of industrial activity [26]. A total of 150,000 tons of residual ash were used as fertilizer in 2007 [27]. It is essential to establish a sustainable ash management plan that includes ash generated by natural processes since the cost of ash disposal keeps rising as a result of the growing amount of ash produced [28]. The most typical method for removing ash is to dump it in a landfill; this method accounts for 70% of all ash production, with the remaining 20% or 10% being used for other uses or as a supplement to the soil [29]. The manufacturing of food and other goods uses the bulk (54%) of the leftover ash. The remaining ash was used for a variety of tasks, such as composting, making soil mixes, replanting damaged regions, and other things [27]. About half of the wood ash resource produced each year is still dumped in landfills, which pollutes the environment.

Wood Ash is the excess powder that remains after burning wood and is discarded from both home and industrial power plants. The residue that remains after burning wood in a wood-fired oven creates WA in the industry [30]. Usually the amount of ash produced by burning wood is 6–10% by weight, and its makeup may vary greatly depending on the environment and industrial operations [26]. In this case, 70% of the ash produced is disposed of using the most common technique, which is landfilling, the remaining 20% is utilized as soil additive, and 10% is used for other purposes [29]. The features of the ash depend on the properties of the biomass, the method of burning, and the location where the ash is collected [31]. Wood ash is potentially hazardous since it mostly consists of small particulate matter that is readily carried into the air by winds and may create respiratory health issues for livings who live close to the dump site or may contaminate groundwater by leaching harmful substances into the water. A sustainable ash management strategy that incorporates the ash within the natural cycles is necessary since the expense of disposing of the ashes is rising and the amount of ashes is growing [28].

Concrete is made more environmentally friendly by using WA in it, turning it into a resource for creating a cementing material substitute that is very effective [32]. Researchers [33] investigated the morphological, chemical, and physical characteristics of WA as a possible pozzolanic material in their investigation of the use of the substance in cement-based composites. The findings show that these ashes may be utilized in medium-strength concrete in lieu of some of the aggregates or cement. Researchers [34,35] conducted experiments that demonstrated the positive outcomes of WA in terms of strength and came to the conclusion that WA is an appropriate material to use as a replacement for cement in the manufacturing of concrete. A study [36] determined an appropriate compressive strength (CS) limit for casting blocks in concrete manufacturing at a 15% optimal dosage of WA that replaces the cement. Abdullahi et al. [37] discovered an ideal replacement rate of 20% and demonstrated that the amount of wood ash increases as the water need rises. A study [38] investigated the effects of replacing cement in concrete with sawdust ash up to 30% by weight and found that composites containing 10% performed well in terms of workability and resistance. A researcher examined the viability of using several kinds of ashes from biomass combustion for building materials [39]. Research that employed WA as a partial replacement in mortars (0, 10, and 20%) came to the conclusion that the substance might help with construction’s sustainability [40]. A study also claimed that the seismic induced damages in reinforced building is mainly due low materials strength and poor properties [41]. Therefore, using wood ash in lieu of cement in blended cement is advantageous from an environmental standpoint as well as creating affordable building materials, resulting in a sustainable relationship.

Brief literature demonstrates that WA can be utilized as a construction material. However, there is a lack of data, and no one can accurately predict how wood ash would affect concrete qualities, which limits its application. As a result, a thorough analysis is needed to compile previous and present developments in the use of woody ash as a building material. 

The objective of this analysis is to compile all relevant data on WA-based concrete. (a) general background and basic information on wood ash, (b) physical and chemical characteristics of WA, (c) the effect of WA on concrete’s fresh qualities, (d) strength characteristics of WA-based concrete, (e) durability aspects of WA-based concrete, and finally microstructure analysis of WA based concrete. The evaluation also identifies the research gap that needs further investigation.

## 2. Physical and Chemical

**Figure 1:**
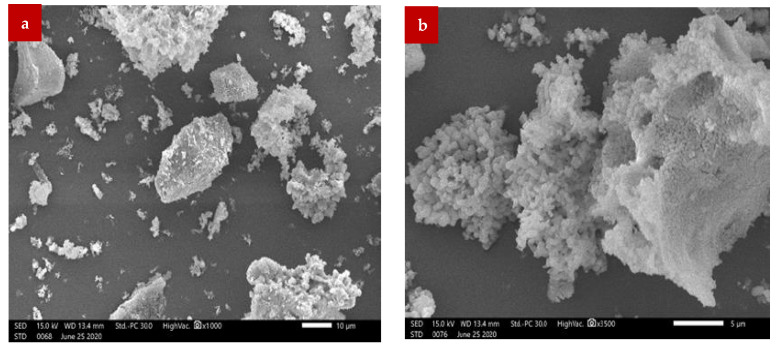
WA Particle SEM: (**a**) 10 µm and (**b**) 5 µm [42].

The wood ash was formed of particles of various sizes and shapes, as seen by the SEM photos. The ash particle had an unusual shape, rough, porous surfaces, and highly uneven particle sizes. The particles’ general arrangement did not follow any pattern as shown in Figure 1. Similar research also came to the conclusion that WA contains fiber and particle phases with smooth and rough surfaces, but that the majority of the shapes were irregular [25].

**Figure 2:**
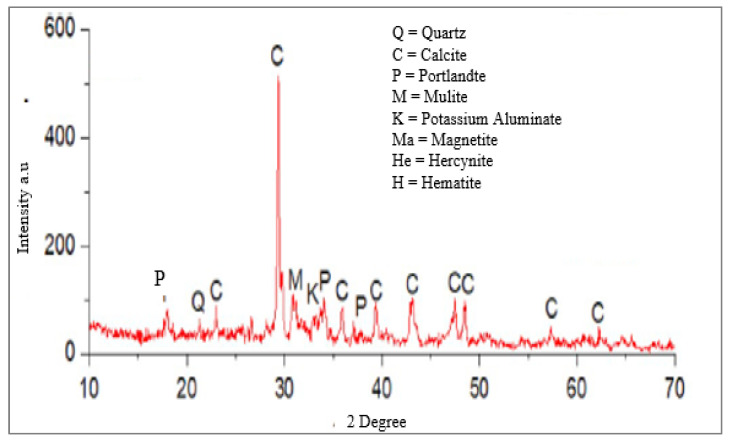
XRD of WA Reprinted from [45] with permission.

Wood ash contains SiO_2_, which indicates that it has an alkaline character, according to several investigations on chemical composition [43]. Additionally, it has a favorable impact on the mineralogy of hydrated cement [44]. The XRD pattern of the WA sample is displayed in Figure 2, which is similar. It displays a hump that indicates it is amorphous as well as SiO_2_ peak representations of its crystalline nature. Thus, it was determined that SiO_2_ exists in both crystalline and amorphous forms in WA. Additionally, a Table 1 with a detailed overview of the chemical makeup of WA according to earlier research is provided. Another claim made in the research was that the pozzolanic activity of the amorphous silica makes it suitable for use as a cement substitute [32].

A pozzolanic reactivity required for effective hydraulic performance was ensured by the substantial levels of silicon dioxide, aluminum oxide, and iron oxide present. Their values, except for calcium oxide, are also equivalent to or even better than those of cement particles. When the proportions of the three oxides are added together, the result is 45.95%, which is below the threshold of 70% required to be labeled as Class F pozzolan [49].

## 3. Fresh Properties

### 3.1. Setting Time

**Figure 3:**
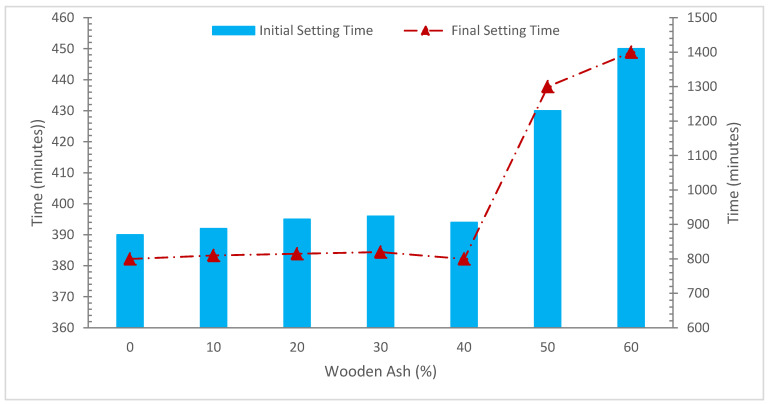
Setting Time: Data Source [47].

Figure 3 illustrates the setting time with varying amounts of WA replacement. It should be mentioned that using WA lengthened the setting time. Ash is less reactive than cement, which causes a delay in cement hydration, which contributes to the prolonged initial and final setting time (IFST) with a rise in pozzolanic material concentration. For civil engineering applications, such as casting deep wells and in certain repair concrete applications, the delay in IFST might sometimes be advantageous [50]. With the WA content in the paste rising from 10 to 30%, it can be seen that the discrepancy between the IFST has also decreased [45].

The IFST in both situations are in line with the numbers specified by the standard TS EN 196-3. This specification states that all mortars must have an initial setting time of at least 60 min and a final setting period of no more than 600 min. The setting of the blended cement made from WA and regular cement was delayed by the addition of the WA instead of cement. This showed that mixed cement paste needed longer IFST. With an rise in the percentage of cement replaced with WA, the consequences of setting time interruptions developed further noticeable [51]. The dilution of cement content when WA was used as an alternative for part of the OPC is the primary cause of prolonged in the IFST of concrete in the existence of WA [51].

WA is less reactive that cement and is included in combined cement paste, causes the cement hydration rate to be delayed, which also delays the combined cement paste setting. A beneficial feature of blended cement is an extended setting time for cement pastes, which implies longer working hours for the paste. WA combined cement paste became appropriate for purposes owing to a correspondingly less hydration heat of the combined cement because of the reduced hydration rate as described before. Due to the required low heat development, stress caused by temperature differences, such as during mass concreting activity, was offset.

### 3.2. Slump Flow

**Figure 4:**
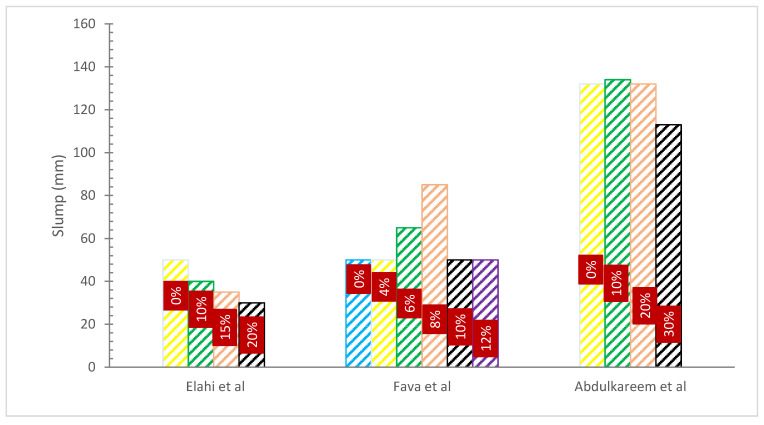
Slump Flow: Data Source [13,45,53].

Figure 4 and Table 2 show the slump flow with various proportions of WA substitution. It should be noticed that WA caused a reduction in concrete’s slump flow. According to studies, production-grade 20 concrete’s ability to function when it was originally mixed suffered by the use of WA from open-flame sawdust burning as a cement replacement component. While maintaining a constant water binder ratio (w/c) of 0.565, the slump value of the concrete mix steadily reduced by 5 to 40 mm when compared to the control concrete mix without any wood waste ash component. At intervals of 5%, the increment level of cement substitution with WA varied from 5% to 30% of the total binder weight [38].

A study examined the effects of adding waste wood ash to concrete in various amounts. It was noted that, given the same w/c, the concrete including WA displayed poorer workability than the control specimen. In addition, when the replacement proportion rose, it rose as well. It was insensitive to a downturn at replacement rates of 20, 25, and 30%. This might have resulted from the ash’s high organic content [52]. The addition of wood waste ash as a partial cement substitute at replacement levels of 10, 20, 30, and 40% resulted in higher water demand of 10, 11.7, 13.3, and 15%, respectively, to obtain similar slump values as the reference slump without the existence of WA [37]. Additionally, it was found that as the amount of cement substitution with wood waste ash increased, the value of the concrete mix’s compacting factor decreased [52].

The flow test showed that after 10 to 30% of cement substitution with wood ash, the mortar mixes’ workability was reduced [54]. The physical characteristics of woody ash, such as its irregular particle form and greater surface area, are what cause it to have a negative impact on the workability of concrete. Less workable concrete was produced because a bigger surface area needed more cement paste for flowability. The internal friction between the components of concrete is further increased by irregular form and bigger surface area, which results in less workable concrete. Despite the fact that some studies indicate that wooden structures increased the workability of concrete, this is likely due to the micro filler material that fills the gaps between the components of concrete, increasing the amount of cement paste that is available for lubrication and making concrete more workable.

## 4. Strength Properties

### 4.1. Compressive Strength (CS)

**Figure 5:**
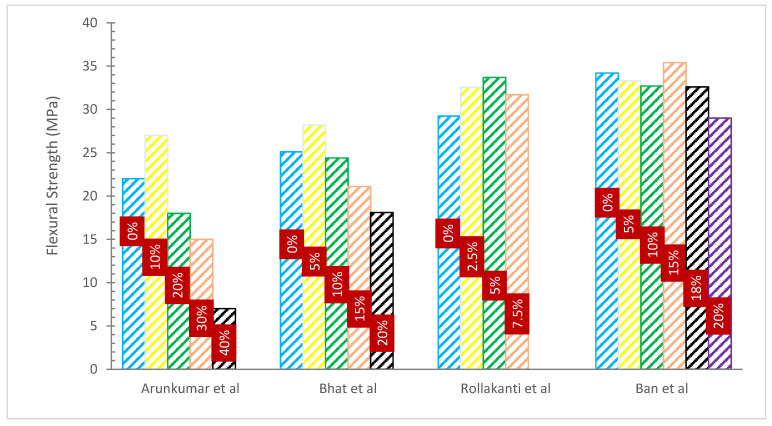
Compressive Strength: Data Source [53,55,56,60].

The CS of concrete with varying percentages of woody ash replacement is shown in the Figure 5 and Table 3. It should be noted that adding wood ash to concrete increased its CS. When compared to typical concrete, the CS of 5% woody ash and seashell after 28 days was at its highest and improved by 15.26%. Later, it was discovered that when the proportion of wood ash and seashell powder was increased, the strength of concrete began to decrease [56]. According to a researcher [51] who examined the development of CS in mortar mixes including wood waste ash as a cement substitute at stepped increments of 5%. The mortar mix containing 10% wood waste ash as a partial cement replacement material showed comparable CS to the same mortar mix with just OPC as binder after a 60-day curing age. Furthermore, The highest CS was seen in mortar mixes with a 10% wood waste ash content at all curing ages up to 60 days [51].

A study also conclude that within hydropathy and sulphate solution, silica fume show more strength as compared to the fly ash at early age [57]. A study conclude that the carbon nanotubes improved the interfacial transition zone which results more strength [58]. The SiO_2_ offered for the WA is bigger than that of cement and has pozzolanic value for little cement substitutes. The silica from the 3% WA may have provided more than was necessary for Ca(OH)_2_ to react, in this instance functioning merely as filler [25]. However, mortar containing 0.5% WWA shows a modest increase in resistance after curing for 90 days, which may be a sign of a pozzolanic interaction between the WA and the cement hydration. The finding is corroborated by the fact that cement hydration is insignificant over prolonged curing intervals [52]. 

In research, the CS of cement mortar mixes including fly ash from wood biomass-fired power plants was examined. Fly ash made from wood waste was utilized to substitute cement at levels of 10, 20, and 30% of the total weight of the binder. When compared to equal plain OPC mortar, mortar mixes containing a 10% wood waste fly ash ingredient showed greater 28-day CS but reduced FS. The 28-day CS of the mortar mix was found to be lower when wood waste fly ash was used as a partial cement replacement material at higher replacement levels of 20 and 30% of the total binder weight [59].

CS test results at 7 and 28 days indicated that cement was used in lieu of 10% wood ash to achieve the highest strength possible [56]. Later, strength metrics are shown to be declining when the proportion of wood ash is raised. Wood ash had a beneficial effect on the mechanical characteristics of concrete as well as its resistance to freezing and thawing [26]. According to research that examined the CS of concrete that had been changed with 0–40% WWA, the ideal replacement rate for cement was found to be 20% [37]. Pozzolanic activity has been researched in concrete and is connected to the creation of extra CSH binder via the interaction of the ash’s SiO_2_ with the cement’s Ca(OH)_2_ and the ash’s CaO content with water [37].

The pozzolanic reaction of wood ash, in which silica present in wood ash reacts with calcium hydrates (CH) formed by the hydration of cement to form calcium silicate hydrates (CSH) gel, has a positive effect on the CS of wood ash on concrete. This gel provides secondary binding properties and increases CS. According to earlier studies, wood ash fills the spaces between the components of concrete, making it denser and increasing CS. However, a larger dosage of wood ash (30%) has a negative impact on concrete’s CS because concrete lacks workability, and increased compaction allows for more pores to form in hardened concrete, which reduces CS [22,32]. Additionally, the CS of the pozzolanic material may be diminished at larger doses owing to the dilution effect, which results in an alkali-silica interaction. As a result, it is suggested that up to 20% of the cement used in the substation’s construction be made of wood ash. In contrast, the research found that with WA substitution of 10%, 20%, and 30%, respectively, the percent drop in the CS was 26, 32, and 30. This drop with an increase in WA content might be attributable to both the use of less cement and the WA’s lack of pozzolanic impact [54]. This decline in CS is acceptable since a particle function more as a filler than as a binder inside the matrix of cement paste. The strength decreases as the replacement % rises because more cement has to be applied to the filler material’s larger surface area. However, the intensity grew with age, indicating the existence of a pozzolanic response [32].

**Figure 6:**
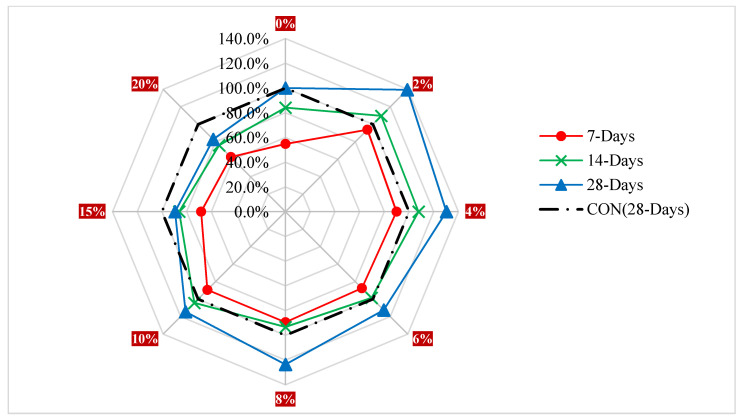
Relative Strength (CS).

Figure 6 illustrates the CS with different amounts of WA at various curing periods. The benchmark strength is calculated at 28 days using the blank mix (control or reference concrete CS). The CS is 6% less than the reference strength after 7 days of curing when WA is used in lieu of the reference material at a 2% replacement rate, but 10 and 40% greater than at 14 and 28 days, respectively. The CS is 11% less than the reference strength after 7 days of curing, but 4 and 15% greater than at 14 and 28 days, respectively, when the replacement rate is 10%. At 7, 14, and 28 days after curing, the CS is 38, 24, and 18% less than the reference strength at a 20% replacement rate. It’s possible that the strength increased with age because the pozzolanic reaction occurred more slowly than the cement hydrated. It may be assumed that adding up to 10% of wood ash to concrete won’t have a detrimental effect on its CS.

### 4.2. Tensile Strength (TS)

**Figure 7:**
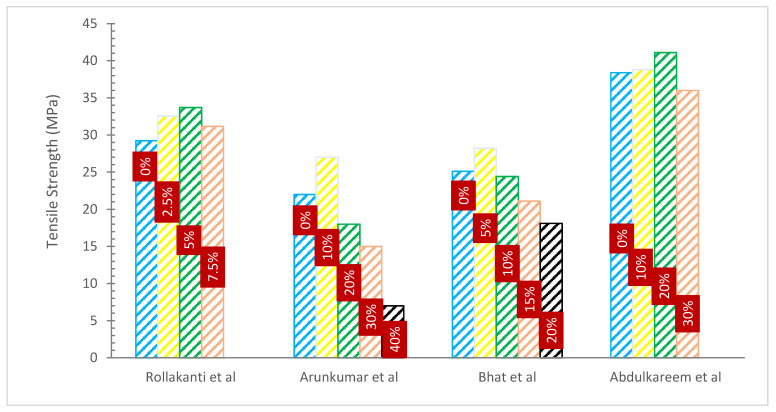
Tensile Strength: Data Source [45,56,58,61].

Figure 7 and Table 4 show the TS of concrete with various percentages of WA substitution. It can be noted that the TS of concrete increased with WA. The pozzolanic reaction, which provides extra binding properties, increased the link between the constituents of concrete (aggregate) and fibers, leading to higher TS. This is the cause of the beneficial impact of woody ash on the TS of concrete. Data analysis reveals that TS of the WA blended cement concrete decreased as WA content increased, however, the decrease was less significant than the decrease in CS. This decrease may be ascribed to the WA particle acting as a filler in the concrete and the WA particle’s poor bonding with the mortar matrix because of its large surface area [32]. A study was conducted on the TS of concrete mixtures that included sawdust ash as a partial substitute for cement at 7 and 28 days. With an increase in saw dust ash %, he observed a loss in TS, however, it was less obvious than a decrease in CS. It was observed that the strength differential between the blended cement concrete and control mixtures increased after 7 days, and after 28 days, blended cement concrete mixes with replacement percentages of up to 25% had TSs of up to 90% of the control mixtures’ strength [34].

When employed as a cement substitute in the manufacturing of concrete, a researcher looked at the influence of WA on the TS of the concrete. The replacement levels of 5%, 8%, and 12% WA was employed. For comparison, a similar reference concrete devoid of WA was poured. The manufactured concrete specimens’ tensile strength was assessed after 3, 7, 28, 91, 182, and 365 days. The TS of control concrete was reported to be 3.8 MPa at 28 days and 4.3 MPa at 365 days from the laboratory results analysis. the TS of concrete mixtures with WA content varied between 3.6 and 4.0 MPa at 28 days and between 4.3 and 5.3 MPa at 365 days. Additionally, it was found that the concrete with a WA of 8% displayed the best TS development behavior for concrete ages over 28 days up to 365 days, with a magnitude of TS that consistently outperformed that of other test mixes [68].The average control TS of concrete is 6.4 MPa after 28 days of curing and rises to 10.8 MPa with a 20% replacement of wood, or about 68% more than reference concrete. Notably, hardwood ash enhanced tensile more efficiently than concrete’s CS [65]. Research, however, asserted that the TS for a 5% mixture of seashells and hardwood ash was maximal and increased by 12.5%. Therefore, when the amount of wood ash and seashell powder in concrete was raised from 0 to 10%, TS improved as a result, and when 15% was substituted, the strength began to decrease by 4.04% [56].

### 4.3. Flexural Strength (FS)

**Figure 8:**
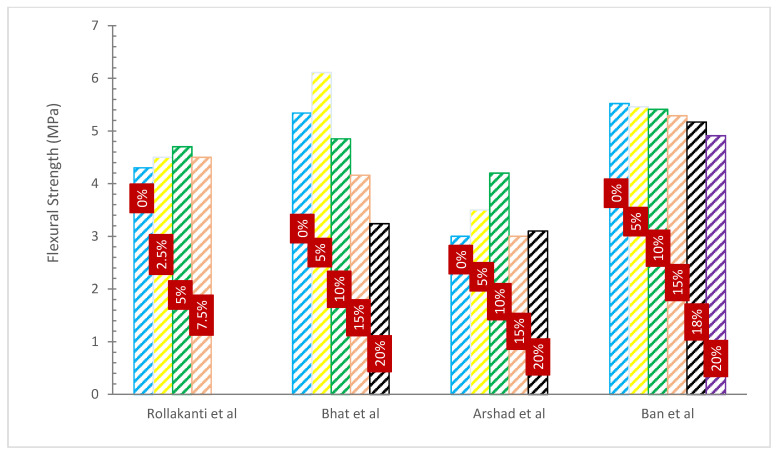
Flexural Strength: Data Source [53,55,56,63].

Figure 8 and Table 5 show the FS of concrete with various percentages of WA substitution. It can be noted that the FS of concrete increased with WA. In comparison to ordinary concrete, FS increased by 7.56% when cement was used to replace 10% of wood ash and seashell powder [56]. The data analysis clearly shows that the usage of WA caused the FS to drop with increasing wood ash content. As the quantity of wood ash increases, more cement is required to cover the filler particle, which results in poor bonding in the matrix and a decrease in the strength characteristics [32].

The research assessed the FS of mortar mixes created by partially substituting fly ash from two different wood biomass power plants for the cement binder. Wood fly ash was used to make mortar bars specimens for flexure testing at concentrations of 0 (control mortar), 10, 20, and 30% by total binder weight. Flexure strength measurements showed that the strength of mortar mixes created with varying percentages of wood fly ash replacing cement, ranging from 0% to 30%, gradually decreased. Mortar mixtures that included 10, 20, and 30% of wood fly ash in place of some of the cement showed FSs that were, respectively, 60.6–71%, 59.6–61.7%, and 45–48.6% more than those of the control mix. They came to the conclusion that the mechanical strength of mortar degraded quickly with the addition of wood waste ash at cement replacement levels greater than 20%, and that wood fly ash can be utilized in the mortar at a replacement level of cement up to 20% [59].

The CS, TS, and FS of concrete made using blended WA cement are tested and assessed in a study. There are two distinct water-to-binder ratios (0.4 and 0.45) and five different replacement percentages of WA (5%, 10%, 15%, 18%, and 20%), as well as control specimens for both water-to-cement ratios, that are taken into consideration. The results of CS, TS, and FS indicated that although strength rose with aging, it only slightly reduced with a rise in wood ash contents. The examination of the study’s findings led to the conclusion that WA may be mixed with cement without impairing the concrete’s strength capabilities [32].

Although more slowly than CS, FS also declined as WA content rose. Samples containing 5% WA had a 28-day FS of 5.20 N/mm^2^, whereas samples with 30% WA had a value of 3.74 N/mm^2^. At equivalent ages and additive amounts, the FS magnitudes of WA concrete varied from 67 to 93% of the control concrete [52].

The research found that up to 20% more wood ash was substituted for sand, slightly enhancing the FS. The maximum FS was reached at a partial replacement of 20% after 28 days with an improvement in performance of 65.46%. Sand replacements of greater than 20% led to a drop in FS. Additionally, the FS has gradually declined when the percentage of wood ash has increased as a partial replacement of cement at a young age; yet, the maximum FS was achieved at a partial replacement of 4% at 28 days, with a performance gain of 5.70% [66].

## 5. Durability

### 5.1. Density

**Figure 9:**
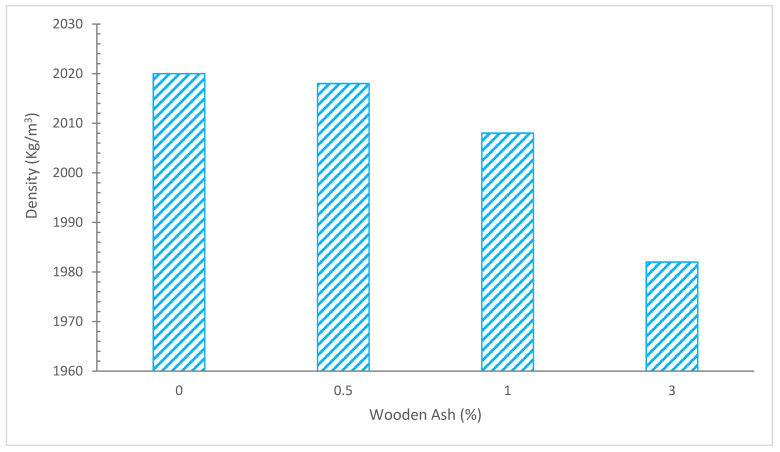
Density: Data Source [25].

Figure 9 displays the values of mortar unit weights as a function of WA content as determined after 90 days of curing. As can be seen, as the quantity of WA is greater, a drop in the relative specific gravity is visible. The lowest densities are obtained by replacing 3% of the cement, which is a drop of 2.72% in comparison to plain mortars. The use of WA has been associated with a reduction in density on concrete previously [69], and this reduction becomes more pronounced at greater replacement levels than those used in our work. This decrease is brought on by the WWA’s reduced specific gravity when compared to regular Portland cement [70]. 

Although mortar with a 3% WWA component has low densities, no combinations can be categorized as lightweights since their maximum density is 1800 kg/m^3^ [71]. A study also conclude that combination of metakaolin and steel fibers improved durability of concrete for road construction [72].

### 5.2. Water Absorption

**Figure 10:**
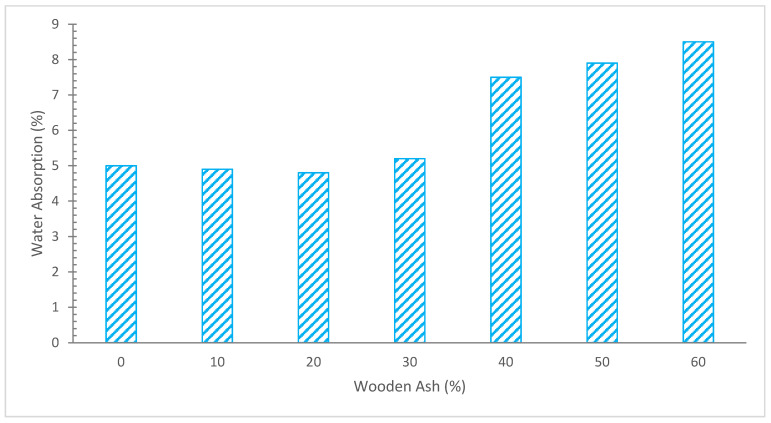
Water Absorption: Data Source [47].

The water absorption rate is shown in Figure 10. With varied replacement percentages of WA. It should be noted that concrete’s water absorption is unaffected by the insertion of up to 20% hardwood ash. The findings show that as the amount of SCM rises, so does the percentage of concrete that absorbs water. Water-absorbing materials are known as WA. Additionally, WA is hydrophilic, which causes it to absorb water at a rate that rises with proportion [47]. 

Similar to this, a study’s conclusion on the concrete’s water absorption capabilities when wood waste ash is used as a partial substitute for cement. For water absorption experiments, concrete mixtures with wood waste ash contents ranging from 5% to 30% were created in 5% increments. With the rise in cement replacement from 5% to 30%, it was found that the water absorption of concrete including wood waste ash as a partial replacement material increased steadily from 0.14 to 1.05% [52].

Using wood waste ash as a partial substitute for cement in mortar mixtures was the subject of a study to see how it affected the mortar’s capacity to absorb water. The identical mortar mix proportions were used to make two batches, one of which had no wood waste ash content and the other of which included a partial replacement material of 15% wood waste ash. The decrease in the amount of water absorbed by the mortar mix generated was discovered to be a result of the use of wood waste ash as a cement replacement ingredient at a total weight of 15%. 0.8% and 1.25%, respectively, were found to be the average water absorption rates of mortar mixtures containing 15% wood waste ash and those without, both of which are still far below the 10% limit [73].

According to research, mortars containing up to 20% WA had lower overall porosity and water absorption at the young ages of 3–7 days than the control mix. This is explained by the fact that the geopolymer structure was improved because of the high amount of reaction products [45]. Low porosity was found for the changed mortars, which may be a sign that concrete is absorbing less water. A likely explanation is that calcium silicate hydrate (C-S-H) amorphous gels filled the matrix’s holes, creating a thick microstructure. This results in a higher C-S-H concentration and a lower proportion of non-hydrated cement [25]. 

The secondary CSH gel is produced by the pozzolanic reaction of wood ash, which increases the binding characteristics and creates a denser matrix with less water absorption. A denser concrete is produced by micro-filling wood ash, which results in less water absorption. However, at higher temperatures, water absorption increased because the concrete was less workable, which created more pores in the hardened material and enhanced water absorption. The previously released statistics on the effect of ash on the water demand of concrete mixes cannot be used to make any firm conclusions since both an increase and a reduction in water demand have been observed.

### 5.3. Acid Resistance

**Figure 11:**
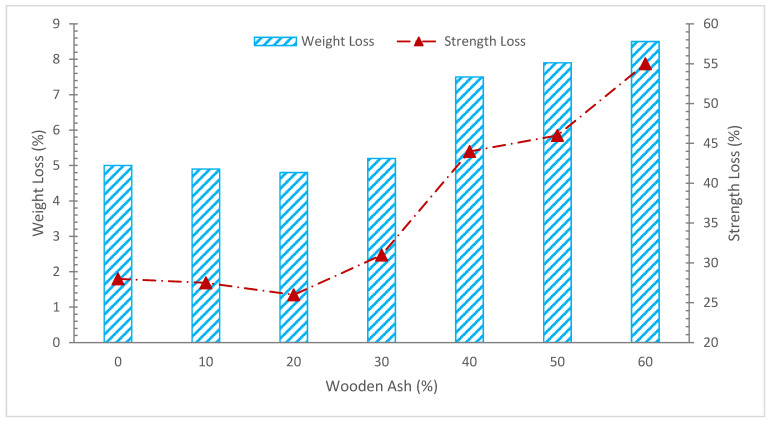
Weight and Strength Loss: Data Source [47].

Figure 11 shows the weight and strength loss of concrete with various percentages of WA when subjected to sulphuric acid. It can be noted that the initial percentage of weight loss decreases as the proportion of wood ash in concrete rises. The weight loss accelerates when the WA percentage rises over 20%.

In research, the acid attack resistance of concrete containing wood waste ash was examined. An identical mix of concrete was used to generate two batches of concrete specimens. One of the batches employed plain OPC as a binder, whereas the other batch used cement and wood waste ash as a partial cement replacement at a ratio of 15% total binder weight to 85% total binder weight. The resulting concrete cubes were then submerged in a 20% nitric acid solution. The cubes were submerged, and at 7, 14, 21, 28, 35, 42, 49, 56, 63, and 70 days, the mass loss was measured. Due to water absorption, both sets of concrete specimens saw a continual marginal increase in mass after being submerged in the strong nitric acid solution for up to nine weeks. It was found that the mass loss of the concrete specimens with 15% of the total weight of wood ash as a binder was less evident in comparison to the control concrete specimens with plain OPC as a binder at the tenth week of immersion [34]. 

Additionally, according to earlier studies, woody ash functions as a micro-filler, filling the spaces between the components of the concrete to produce thick concrete that is more acid resistant. However, a larger dosage of woody ash (30%) has a negative impact on acid resistance because it reduces the workability of concrete and causes more holes in cured concrete, which reduces acid resistance [74].

## 6. Microstructure Analysis

### 6.1. Scan Electronic Microscopy (SEM)

**Figure 12:**
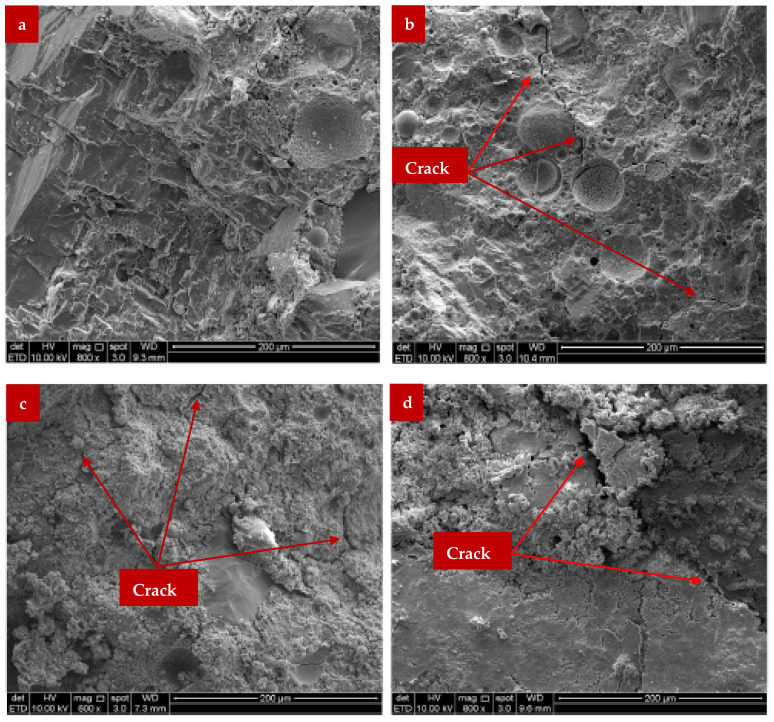
(**a**) 0%,(**b**) 10%,(**c**) 20% and (**d**) 30% WA [45] Used as per Elsevior Permission.

Figure 12 shows the micrographs of the mortars at 28 days curing. By using the quality of the resulting matrix, it is possible to see that the control mixture’s microstructure appearance (Figure 12a) has greatly improved from its original microstructure at 3 days of age. Compared to the starting matrix, the final matrix after 28 days seems more uniform and denser. It is thought that the sequential dissolution and polycondensation of the unreacted/partially reacted fly ash microspheres over time led to the creation of this microstructure [36]. The content of the unreacted/partially reacted microspheres dropped significantly, supporting this. In comparison to its initial characteristics reported at age 3, the control mix had better strength and porosity characteristics at age 28 due to its high microstructural refinement.

At age of 28 days, a mixture of 10% WA still exhibits microstructure traits of a strongly reactive geopolymeric matrix. A thick and continuous geopolymeric matrix with little microcrack content is shown in Figure 12b. On the other hand, Figure 12c shows a severe degradation in the microstructure of the mixture with 20% hardwood ash after 28 days of continuous aging. The resulting matrix is composed of a loose structure with many high-microcracks and precipitated agglomerated products on its surface. Given that the combination includes less fly ash than the control mixes roughly 20% less—the degradation in the microstructure of the 20% WA mix is acceptable. Therefore, with extended aging of 28 days, inadequate Si and Al species restrained the development of geopolymeric gels. Additionally, as shown in Figure 12, the 28-day extension of aging seems to have no impact on the microstructure development of the 30 woody ash mix. This demonstrates the unsatisfactory geopolymerization response after the substitution of high WA content (about 30%) for fly ash. A study also observed that the calcium carbonate generated by microorganisms in the cavities was the key element [75].

A microstructural examination of concrete mixtures comprising 0% and 10% by total binder weight of wood waste ash revealed a considerable decrease in the porosity of cured mortar for the later concrete mixture. Furthermore, it was discovered that adding 10% sawdust ash in place of ordinary Portland cement (OPC) when creating concrete mix significantly decreased the amount of non-hydrated cement and portlandite while significantly increasing the amount of CSH gel present in the concrete mix after a specific curing age [76].

**Figure 13:**
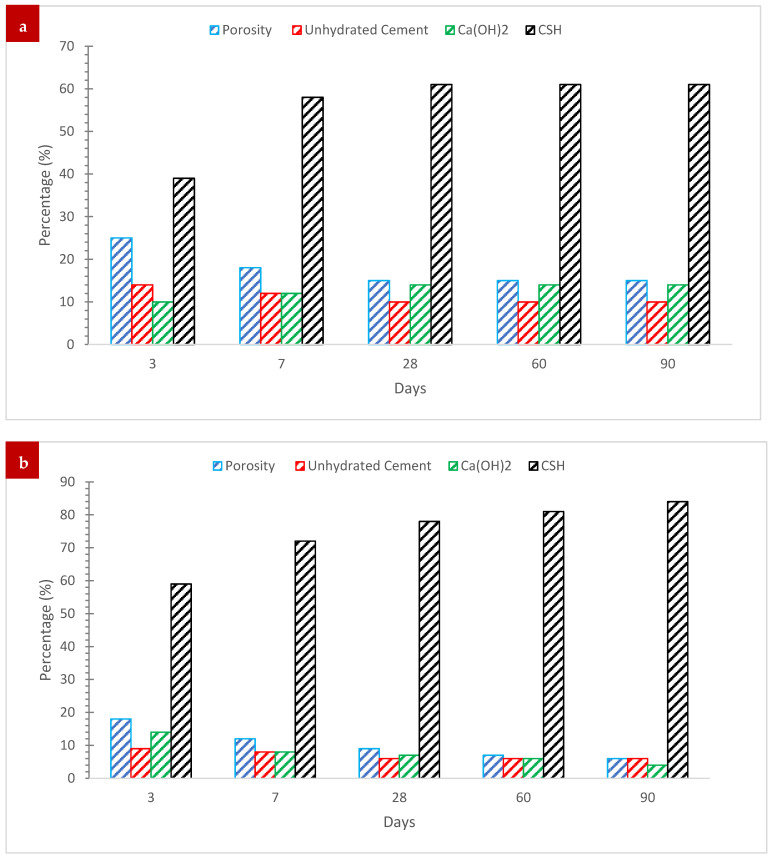
(**a**) Control and (**b**) 10% Sawdust Ash [76].

Pozzolanic reactions were shown to persist beyond the hydration age of 28 days up to 90 days, producing extra CSH gels in the concrete mix with 10% sawdust ash of the total binder weight. Figure 13a shows that after 28 days of curing, the synthesis of CSH gel in an equivalent concrete mix containing plain OPC without sawdust ash content had essentially ceased, as seen by the stationary quantity of CSH gel. Continuous production of CSH gel within a concrete mixture containing 10% sawdust ash as display in Figure 13b helped to densify the cement paste matrix’s microstructure, reduce the amount of mix porosity, enhance the cement-aggregate interfacial transition zone quality, and improve the uniformity of pore distribution within the cement paste matrix. The mechanical strength and longevity of the resulting hardened concrete mix were improved by these improvements in the microstructural characteristics of the cement paste matrix.

### 6.2. Isothermal Calorimetry and Le Chartiler Expansion

**Figure 14:**
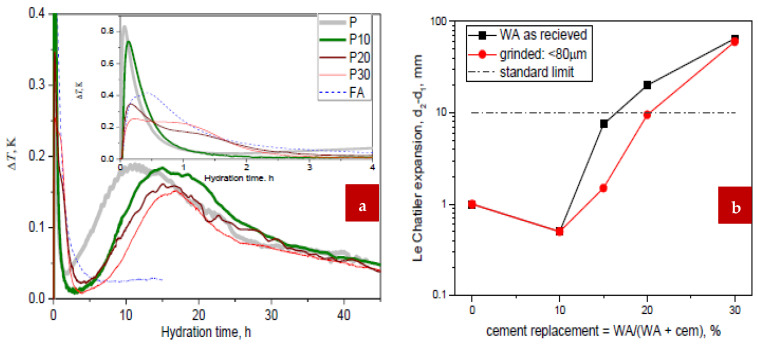
(**a**) Isothermal Calorimetry and (**b**) Le Chartiler Expansion [77].

According to a study of the calorimetric data in Figure 14a, samples with a higher concentration of woody ash experienced the end of the induction phase after 2, 5, 6, and 7 h, respectively. The reaction entered the induction stage immediately after the hydration of pure cement, which produced the largest and narrowest initial heat maximum. Woody ash, on the other hand, exhibited an extended initial hydration maximum and no clearly visible induction phase, although the processes of dissolution and precipitation overlapped. There were two initial maximums stated after the addition of woody ash. When compared to the second, later one (at 1 h), the first, earlier one (at 0.1 h) was lowered. Ash was added, which resulted in a delay in the time needed for the mixture to set and a lower primary maximum of the reaction rate that was achieved at later periods (11, 15, 16, and 17 h), as well as a significant delay in the hydration of the cement. With additions of (10, 20, and 30%) ash, the final heat developed after 45 h was dramatically reduced by -6%, -13%, and -25% of the reference cement, respectively, due to the slowing of the hydration processes. The hydration processes of CaO and MgO, as well as larnite and aluminate phases, are responsible for the considerable hydration heat generation seen in the hydration of ash alone.

The results of tests for volume stability and soundness According to Figure 14b, adding more than 15% of ash causes unwanted expansions (the maximum value is 10 mm), which rise quickly when more ash dosage is added. It is intriguing that, as validated by three mixing repeats of the test with a total of nine duplicates, the 10% addition exhibits less expansion than plain cement paste. For hydration of ordinary ash, the expansion of 70 mm was seen even without boiling after 24 h of curing. Washing (pre-hydration and carbonation) and mechanical (grinding) pre-treatments may reduce or even completely eliminate the negative effects of this expansion.

## 7. Conclusions

To enhance sustainability in construction, research on industrial waste and alternative building materials has focused on substituting some of the cement in concrete. Wood ash is one such substance. The paper examines how timber ash affects concrete characteristics. The analysis leads to the following finding.

- The chemical composition of WA is similar to that of ordinary cement. Therefore, it can be used as a cement substitute in concrete.
- The setting time of concrete increased with the substitution of WA due to pozzolanic activity.
- The workability of concrete declined with the substitution of WA due to the larger surface area and irregular particles of WA.
- Strength and durability of concrete enhanced with WA due to combined pozzolanic activity and micro filling effects of WA. However, higher dose results decreased in strength and durability of concrete due to a lack of flowability which more pores in hardened concrete. Different researchers suggest different optimum percentages of WA due to the change in the source of WA. However, the majority of researcher suggests a 10% optimum substitution of WA.
- Scan electronic microscopy results reveal that WA improved the microstructure of concrete due to pozzolanic activity and micro-filling pores.

## 8. Recommendation

- Although the chemical composition of WA is similar to that of cement, but less research focuses on WA as cement substitution. A more detailed investigation is required in this area.
- No or less information is available on permeability, acid resistance, dry shrinkage and creeps’ properties. Therefore, details research is required in this area.
- The thermal properties of WA-based concrete should be explored.
- More details investigations are required on thermogravimetric, and Fourier transform infrared
- The environmental advantages of WA concrete through life cycle assessment should be explored.

## Figures and Tables

**Table 1:** Chemical Composition of WA.

Reference	[46]	[47]	[13]	[48]	[25]
SiO_2_	48.96	25.0	55.52	2.70	29.1
Al_2_O_3_	11.24	0.76	3.11	1.30	10.3
Fe_2_O_3_	0.6	2.76	0.41	1.30	6.55
MgO	5.05	1.76	2.32	8.70	4.7
CaO	11.59	35.60	9.92	61.0	35.6
Na_2_O	8.76	2.0	-	-	1.72
K_2_O	5.42	3.20	-	12.0	2.91

**Table 2:** Slump Flow of Concrete.

Reference	WA Substitution Rate	Additional Materials	W/C	Optimum Dose	Slump (mm)	Remarks
[13]	0%, 10%, 15% and 20%	-	0.60	-	50, 40, 35 and 30	Declined
[48]	0%, 10%, 20%, 30%, 40%, 50%, 60%, 70%, 80%, 90% and 100%	Blast Furnace Slag	0.44	50%	160, 150, 155, 150, 150, 145, 145, 150, 145, 150 and 150	No effects
[53]	0%, 10%, 15% and 20%	-	0.45	10%	680, 650, 660 and 640	Declined
[45]	0%, 10%, 20% and 30%	-	-	10%	132, 134, 132 and 133	Improved
[55]	0%, 4%, 6%, 8%, 10%, 12% and 16%	-	0.32	8%	50, 50, 65, 85, 50, 50 and 90	Improved

**Table 3:** Compressive Strength of Concrete.

Reference	WA Substitution Rate	Additional Materials	W/C	Optimum Percentage	Compression Strength (MPa)	Remarks
[46]	0%, 5%, 10%, 15%, 20% and 25%	_	0.60	_	7 Days 10, 03, 04, 10, 11 and 08 14 Days 12, 05, 14, 11, 16 and 09 21 Days 15, 09, 15, 08, 12 and 10 28 Days 20, 09, 14, 12, 13 and 09	Declined
[56]	0%, 2.5%, 5% and 7.5%	_	0.45	5%	7 Days 18.96, 21.28, 22.45 and 19.64 14 Days 26.14, 29.32, 30.41 and 27.93 28 Days 29.23, 32.54, 33.69 and 31.17	Improved
[47]	0%, 10%, 20%, 30%, 40%, 50% and 60%	Seashell Powder	0.45	20%	3 Days 35, 36, 40, 35, 32, 25 and 22 7 Days 55, 58, 60, 52, 45, 38 and 35 28 Days 42, 43, 45, 35, 33, 25 and 22	Improved
[61]	0%, 10%, 20%, 30 and 40%	_	0.50	10%	7 Days 15, 17, 12, 07 and 04 14 Days 19, 22, 15, 10 and 06 28 Days 22, 27, 18, 15 and 07	Improved
[13]	0%, 10%, 15% and 20%	_	0.60	-	7 Days 23.5, 21.2, 16.9 and 21.0 28 Days 30.0, 29.5, 23.5 and 15.4	Declined
[48]	0%10%, 20%, 30%, 40%, 50%, 60%, 70%, 80%, 90% and 100%	Blast Furnace Slag	0.44	50%	28 Days 25, 28, 30, 31, 33, 38, 33, 31, 35 and 15 90 Days 28, 35, 37, 39, 40, 44, 39, 39, 50 and 20	Improved
[62]	0%, 5, 10%, 15% and 20%	_	-	5%	7 Days 16.0, 17.2, 15.2, 12.2 and 10.5 28 Days 25.1, 28.2, 24.4, 21.1, 18.1 56 Days 28.2, 29.6, 24.5, 20.6 and 18.3 90 Days 30.4, 31.2, 27.8, 25.3 and 21.4	Improved
[53]	0%, 10%, 15% and 20%	_	0.45	10%	28 Days 27, 29, 25 and 22	Improved
[36]	0%, 10%, 15%, 20% and 25%	_	-	10%	7 Days 1.95, 2.29, 2.19, 1.97 and 1.19 14 Days 2.43, 2.24, 2.50, 2.62 and 1.19 28 Days 3.10, 2.61, 3.66, 2.80 and 1.40	Improved
[63]	0%, 5%, 10%, 15% and 20%	_	-	-	7 Days 12, 09, 09, 10 and 08 28 Days 16, 15, 15, 13 and 12	Declined
[64]	0%, 10%, 20%, 30%, 40%, 50%, 60%, 70%, 80%, 90% and 100%	Coal Fly Ash	0.30	50%	28 Days 04, 07, 14, 24, 30, 25, 24, 24, 18, 10 and 06	Improved
[45]	0%, 10%, 20% and 30%	_	-	10%	3 Days 32, 32, 32 and 12 7 Days 31, 40, 33 and 13 14 Days 55, 60, 30 and 18	Improved
[25]	0%, 0.5%, 1% and 3%	_	0.40	0.5%	7 Days 23, 26, 24 and 23 28 Days 36, 35, 34 and 33 90 Days 41, 42, 37 and 32	Improved
[65]	6%, 12%, 18%, 24% and 30%	_	-	30%	3 Days 16, 17, 18, 19 and 20 7 Days 25.5, 26.0, 26.5, 27.0 and 27.5 28 Days 34, 35, 36, 37 and 38	Improved
[54]	0%, 10%, 20% and 30%	_	0.4	20%	7 Days 32.5, 35.0, 32.3 and 26.6 28 Days 38.4, 38.8, 41.1 and 36.0 90 Days 39.0, 40.0, 41.2 and 37.7	Improved
[55]	0%, 4%, 6%, 8%, 10%, 12% and 16%	_	0.32	8%	7 Days 50, 51, 53, 54, 48, 49 and 50 28 Days 38, 42, 43, 49, 46, 42 and 44	Improved
[32]	0%, 5%, 10%, 15%, 18% and 20%	_	0.4 to 0.45	-	7 Days 33.0, 31.1, 30.7, 32.3, 30.1 and 27.7 28 Days 34.2, 33.3, 32.7, 35.4, 32.6 and 29.0	Declined
[66]	0%, 2%, 4%, 6%, 8%, 10%, 15% and 20%	_	0.68	2%	7 Days 11.4, 19.5, 18.7, 18.2, 18.6, 18.6, 14.2 and 13.0 14 Days 17.5, 22.8, 22.4, 20.5, 19.4, 21.7, 17.9 and 15.8 28 Days 20.8, 29.0, 27.1, 23.4, 25.7, 23.8, 18.6 and 17.2	Improved
[67]	100%, 70%, 60% and 50%	Fly Ash	0.35	60%	7 Days 6.010, 11 and 6.0 90 Days 9.0, 15, 13 and 13	Improved

**Table 4:** Tensile Strength of Concrete.

Reference	WA Substitution Rate	W/C	Optimum Percentage	Tensile Strength (MPa)	Remarks
[56]	0%, 2.5%, 5% and 7.5%	0.45	5%	28 Days 2.64, 2.76, 2.97 and 2.85	Improved
[61]	0%, 10%, 20%, 30 and 40%	0.50	10%	7 Days 2.6, 2.7, 2.4, 2.2 and 2.0 14 Days 3.0, 3.1, 2.7, 2.5 and 2.0 28 Days 3.3, 3.4, 3.0, 2.9 and 2.4	Improved
[62]	0%, 5, 10%, 15% and 20%	-	5%	7 Days 1.98, 2.14, 1.86, 1.74 and 1.62 28 Days 2.25, 2.56, 2.09, 1.76 and 1.74 90 Days 2.72, 2.92, 2.85, 2.48 and 2.39	Improved
[53]	0%, 10%, 15% and 20%	0.45	10%	28 Days 2.36, 2.72, 2.36 and 1.80	Improved
[63]	0%, 5%, 10%, 15% and 20%	-	-	7 Days 1.1, 0.9, 0.9, 0.9 and 0.6 28 Days 1.5, 1.4, 1.3, 1.3 and 1.2	Declined
[65]	6%, 12%, 18%, 24% and 30%	-	30%	3 Days 1.4, 1.4, 1.4 and 1.5 7 Days 1.5, 1.6, 1.6 and 1.7 28 Days 3.5, 3.6, 3.6 and 3.7	Improved
[32]	0%, 5%, 10%, 15%, 18% and 20%	0.4 to 0.45	-	7 Days 2.50, 2.47, 2.39, 2.27, 2.09 and 2.10 28 Days 3.30, 3.24, 3.16, 3.04, 2.89 and 2.67	Declined

**Table 5:** Flexural Strength.

Reference	WA Substitution Rate	Additional Materials	W/C	Optimum Percentage	Flexure Strength (MPa)	Remarks
[56]	0%, 2.5%, 5% and 7.5%		0.45	5%	28 Days 4.3, 4.5, 4.7 and 4.5	Improved
[61]	0%, 10%, 20%, 30 and 40%		0.50	10%	7 Days 2.7, 3.0, 2.9, 2.5 and 2.1 14 Days 3.0, 3.3, 3.1, 2.9 and 2.3 28 Days 3.3, 3.2, 3.3, 3.0 and 3.0	Improved
[62]	0%, 5, 10%, 15% and 20%		-	5%	7 Days 3.92, 4.24, 3.38, 3.02 and 2.75 28 Days 5.34, 6.11, 4.85, 4.16 and 3.24 56 Days 6.75, 7.03, 6.18, 5.62 and 5.11 90 Days 7.24, 7.84, 6.96, 6.34 and 6.13	Improved
[63]	0%, 5%, 10%, 15% and 20%		-	-	7 Days 2.5, 2.3, 3.0, 2.5 and 2.7 28 Days 3.0, 3.5, 4.2, 3.0 and 3.1	Declined
[64]	0%, 10%, 20%, 30%, 40%, 50%, 60%, 70%, 80%, 90% and 100%	Coal Fly Ash	0.30	50%	28 Days 0.2, 0.4, 0.7, 1.5, 1.7, 1.7, 1.6, 1.5 1.2 and 0.8 365 Days 1.7, 0.6, 0.7, 1.7, 2.3, 2.6, 2.3, 2.2, 2.0, 1.9, 1.5 and 1.0	Improved
[65]	6%, 12%, 18%, 24% and 30%		-	30%	3 Days 4.7, 4.8, 4.9, 5.0 and 5.0 7 Days 4.6, 4.7, 4.8, 4.9 and 5.0 28 Days 6.1, 6.2, 6.3, 6.4 and 6.5	Improved
[32]	0%, 5%, 10%, 15%, 18% and 20%		0.4 to 0.45	-	7 Days 5.10, 5.08, 4.93, 4.87, 4.84 and 4.77 28 Days 5.52, 5.46, 5.41, 5.29, 5.17 and 4.91	Declined
[66]	0%, 2%, 4%, 6%, 8%, 10%, 15% and 20%		0.68	2%	7 Days 3.59, 2.12, 2.23, 2.15, 2.07, 2.45, 1.67 and 1.44 28 Days 5.24, 3.21, 2.45 2.76, 2.42, 2.62, 2.57 and 2.07 56 Days 5.85, 3.66, 3.71, 2.56, 2.54, 2.20, 2.50 and 1.88	Improved
[67]	100%, 70%, 60% and 50%	Fly Ash	0.35	60%	7 Days 0.6, 1.2, 1.2 and 0.6 28 Days 0.7, 1.6, 1.4 and 1.3 90 Days 1.1, 1.7, 1.5 and 1.5	Improved

## Data Availability

All the data available in main text.
